# A Ratiometric and Colorimetric Hemicyanine Fluorescent Probe for Detection of SO_2_ Derivatives and Its Applications in Bioimaging

**DOI:** 10.3390/molecules24214011

**Published:** 2019-11-05

**Authors:** Yan-Hong Qin, Xiao-Yi Jiang, Yuan-Fang Que, Jing-Yi Gu, Tong Wu, Ayinazhaer Aihemaiti, Ke-Xin Shi, Wen-Yu Kang, Bi-Ying Hu, Jin-Shuai Lan, Yue Ding, Tong Zhang

**Affiliations:** 1 School of Pharmacy, Shanghai University of Traditional Chinese Medicine, Shanghai 201203, China; 15002120171@163.com (Y.-H.Q.); Joy213_8327@163.com (X.-Y.J.); qyf2016here@163.com (Y.-F.Q.); q771577634@163.com (J.-Y.G.); 13501919930@163.com (T.W.); Aynazar0601@163.com (A.A.); Skx_gulala93@163.com (K.-X.S.); kangwenyu274@163.com (W.-Y.K.); hby4477jy@163.com (B.-Y.H.); dingyue1640@shutcm.edu.cn (Y.D.); 2 Experiment Center of Teaching & Learning, Shanghai University of Traditional Chinese Medicine, Shanghai 201203, China

**Keywords:** SO_2_ derivatives, ratiometric fluorescent probe, bioimaging

## Abstract

Based upon the intramolecular charge transfer (ICT) mechanism, a novel ratiometric fluorescent probe **EB** was developed to detect SO_3_^2−^/HSO_3_^−^. The probe displayed both colorimetric and ratiometric responses toward SO_3_^2−^/HSO_3_^−^. It displayed a quick response (within 60 s), good selectivity and high sensitivity (a detection limit of 28 nM) towards SO_3_^2−^/HSO_3_^−^. The SO_3_^2−^/HSO_3_^−^ sensing mechanism was confirmed as the Michael addition reaction by ESI-MS. Moreover, the probe could be applied to measure the level of sulfite in real samples, like sugar and chrysanthemum, and it could also be used to detect SO_3_^2−^/HSO_3_^−^ in HepG2 cells through confocal fluorescence microscopy, which proved its practical application in clinical diagnosis.

## 1. Introduction

Sulfur dioxide (SO_2_), as one of the most serious atmospheric pollutants, can enter the living organisms through the respiratory system and then can be turned into sulfite (SO_3_^2−^)/bisulfate (HSO_3_) in aqueous [1,2,3]. Although these endogenous derivatives in organisms have some functions for relaxing the vascular smooth muscle and resisting oxidation [4,5,6], SO_2_ could cause diseases in the respiratory system, the nervous system and the cardiovascular system, such as strokes, asthmatic attacks, migraine headaches, allergic reactions and ischemic heart diseases [7,8,9,10]. Considering the relationship between SO_2_ and the above-mentioned potential health problems, many countries have enforced strict regulations of the concentrations of SO_3_^2−^/HSO_3_^−^ in food, beverages and medicine. Therefore, effective analytical methods were developed to test the SO_3_^2−^/HSO_3_^−^ concentrations in vitro and in vivo, which is essential for the accurate determination of the biological activity of SO_2_ derivatives [11,12].

Compared with traditional detection methods like titration [13], electrochemistry [14,15] and capillary electrophoresis [16,17], fluorescent probes have strengths in operation, sensitivity and selectivity, and can be applied in real-time bioimaging in vivo. Fluorescent probes based on the (*E*)-1,1,3-trimethyl-2-styryl-1*H*-benzo[e]indol-3-ium platform are popular because of their strong fluorescence, good water solubility and relatively large Stokes shift. For example, based on the skeleton (*E*)-1,1,3-trimethyl-2-styryl-1*H*-benzo[e]indol-3-ium with acetyl substitution, a probe reported by Zhou et al. can usefully test carboxylesterase [18]. Nowadays, there are some fluorescent probes based on different reaction mechanisms to detect SO_3_^2−^/HSO_3_^−^, such as 1,4-Michael addition [19,20,21,22], nucleophilic 1,2-addition [11,23] and deprotection of levulinate [24]. However, most fluorescent probes were based on limitedly single fluorescence changes, which will be influenced due to fluctuations in the system and the probe’s concentration. For example, a probe reported by Yu et al. can usefully test SO_3_^2−^/HSO_3_^−^, but a single fluorescence change was used, which limited its use [25]. While the ratiometric fluorescent probes can gain more accurate results for its capacity to detect fluorescence emission/excitation intensities at two different wavelengths [26,27]. Therefore, compared with the single fluorescent probes, it is evitable for the ratiometric fluorescent probes to be influenced by the environmental changes [28]. Based on the ratiometric fluorescent probes, a probe with CF_3_ substitution reported by Sun et al. can usefully test SO_3_^2−^/HSO_3_^−^, but the probe also reacted with CN^−^, which was a low selectivity for its practical applications [19]. In order to develop new fluorescent probes for SO_3_^2−^/HSO_3_^−^ detection [29], we paid more attention to developing a ratiometric probe that could satisfy the practical applications.

**Scheme 1:**
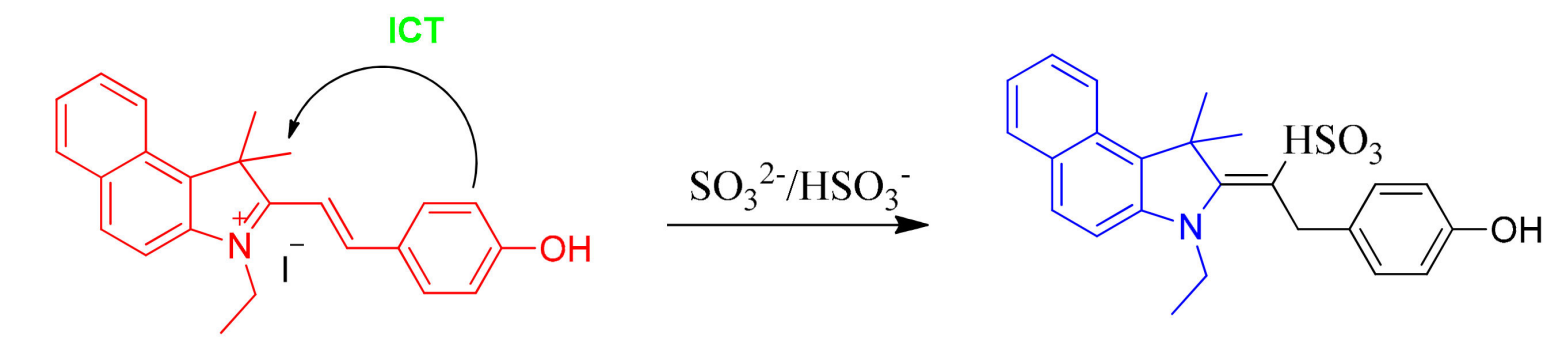
Probe **EB** as a new ratiometric fluorescence probe for sensing of sulfite/bisulfite.

**Scheme 2:**
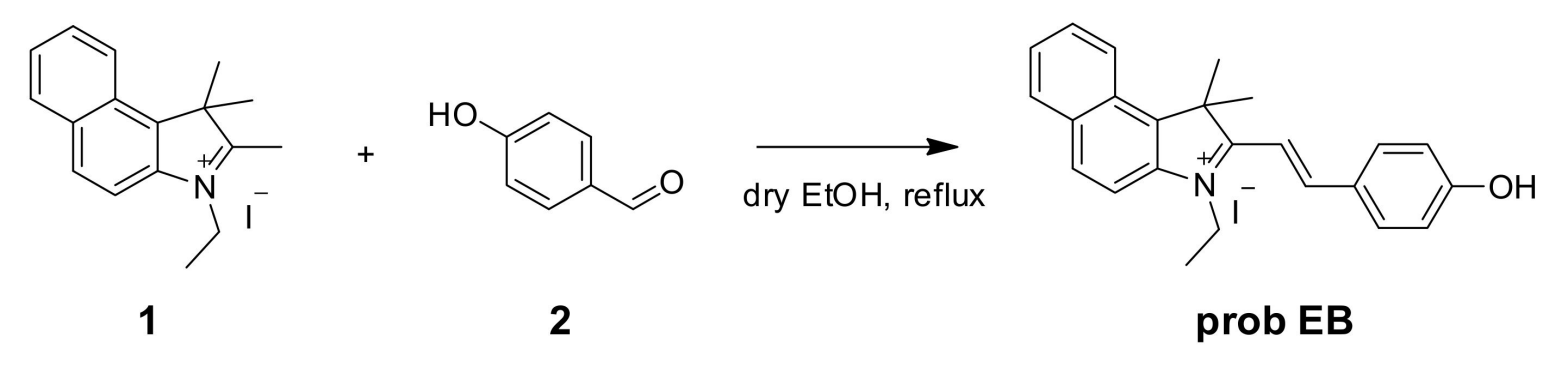
Synthesis of probe **EB**.

On account of the ideas above, we first developed a novel ratiometric fluorescent probe **EB** (*E*)-3-ethyl-2-(4-hydroxystyryl)-1,1-dimethyl-1*H*-benzo[e]indol-3-ium iodide for sensing SO_3_^2−^/HSO_3_^−^ (Scheme 1), which is based on the nucleophilic reaction between SO_3_^2−^/HSO_3_^−^ and the C=C bond. The 3-ethyl-1,1,2-trimethyl-1*H*-benzo[e]indol-3-ium iodide (the hemicyanine part) and 4-hydroxybenzaldehyde were used to synthesize the probe as the intramolecular charge transfer (ICT) donor and the acceptor respectively [30,31]. The hemicyanine was proved to be the effective fluorophores in designing fluorescent chemosensors, which had a blue fluorescence with high quantum efficiency and the chemical stability [20,32,33,34]. After the specific 1,4-addition reaction of SO_3_^2–^/HSO_3_^–^ with the vinyl group of probe **EB** (Scheme 1), the structure of probe **EB** was destroyed by SO_2_ derivatives. Therefore, because of the obvious emission between probe **EB** and the hemicyanine derivative, we could detect two well-resolved emission bands before and after addition of SO_3_^2–^/HSO_3_^–^. Thus, we could observe significant changes in the UV-Vis absorption spectra and fluorescence spectra and make the colorimetric and fluorogenic detection of SO_3_^−^/HSO_3_^2−^ possible. Scheme 2 showed the synthesis route of probe **EB** and the structure of probe **EB** was confirmed by ^1^H-NMR, ^13^C-NMR and MS (Figures S1–S3). What’s more, the probe showed excellent characteristics including the large hypsochromic shift, the ultrafast response time (within 60 s), high sensitivity (a detection limit of 28 nM), low cytotoxicity and excellent selectivity of SO_2_ derivatives over other anions and biothiols. Additionally, it could effectively monitor SO_2_ derivatives and successfully applied in real samples and living cells.

## 2. Results and Discussion

### 2.1. UV-Vis and Fluorescence Spectra for Sulphite Detection

**Figure 1:**
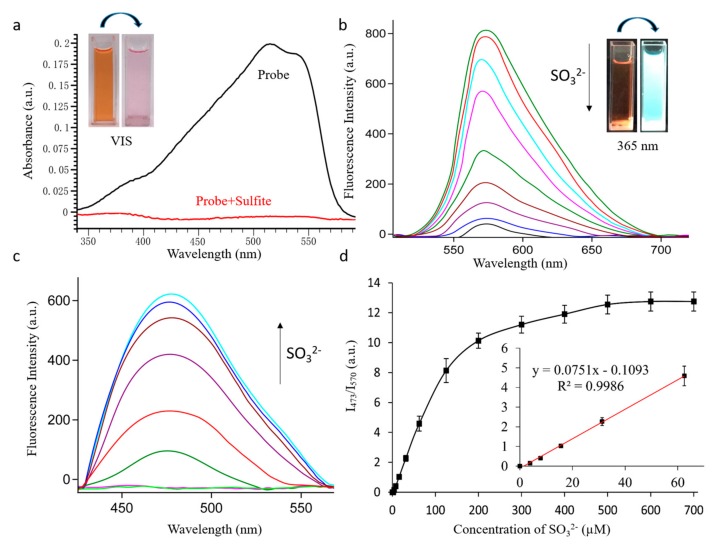
(**a**) UV-Vis absorption spectra changes and (**b**–**c**) fluorescence spectra changes of the probe **EB** (10 µM) in PBS solution (pH 7.4) with increasing amount of SO_3_^2−^ (0–500 µM). For (**b**) λ_ex_ = 485 nm slits: 10.0/10.0 nm; for (**c**) λ_ex_ = 380 nm slits: 5.0/5.0 nm. (**d**) Linear correlation between the emission intensity ratio (I_473_/I_570_) and the concentration of SO_3_^2−^.

First, the UV-Vis absorption spectra of the probe were detected in PBS solution (pH = 7.4). As shown in Figure 1a, the UV-Vis absorption of probe **EB** showed an intense absorption peak at 510 nm, while the absorbance at 510 nm decreased dramatically after it had reacted with 500 µM SO_3_^2−^. At the same time, there was a significant color change: the obvious pink color of solution faded to colorless (Figure 1a and Figure 1a inset), which suggested that a naked-eye colorimetric method could be used expediently to detect SO_3_^2−^/HSO_3_^−^. Next, due to the strong intramolecular charge transfer (ICT), probe **EB** (10 μM) showed a strong fluorescence peak at 570 nm (excitation at 485 nm), while with the increase of SO_3_^2−^(0–500 μM), the fluorescence emission band decreased dramatically at 570 nm (Figure 1b) and a new emission band simultaneously increased at 473 nm (excitation at 380 nm) (Figure 1c). The fluorescent quantum yield (Φ = 0.029) of probe **EB** at 570 nm and the fluorescent quantum yield (Φ = 0.537) of probe **EB** after reaction with 500 μM SO_3_^2−^ at 473 nm has also been determined, with Rhodamine B as a reference. These changes were attributed to the transformation of the conjugated structure of the probe by reacting with SO_3_^2−^/HSO_3_^−^. What’s more, there was a linear correlation between the fluorescence intensity ratios (I_473_/_570_) and the concentration of SO_3_^2−^ ranging from 0 to 60 µM (Figure 1d). According to the signal to noise ratio (S/N = 3), the limit of detection came out to be 28 nM, suggesting probe **EB** could be applied to detect traces of SO_3_^2−^ under environmental or biological conditions.

### 2.2. The Selective Response of Probe to Sulfite

**Figure 2:**
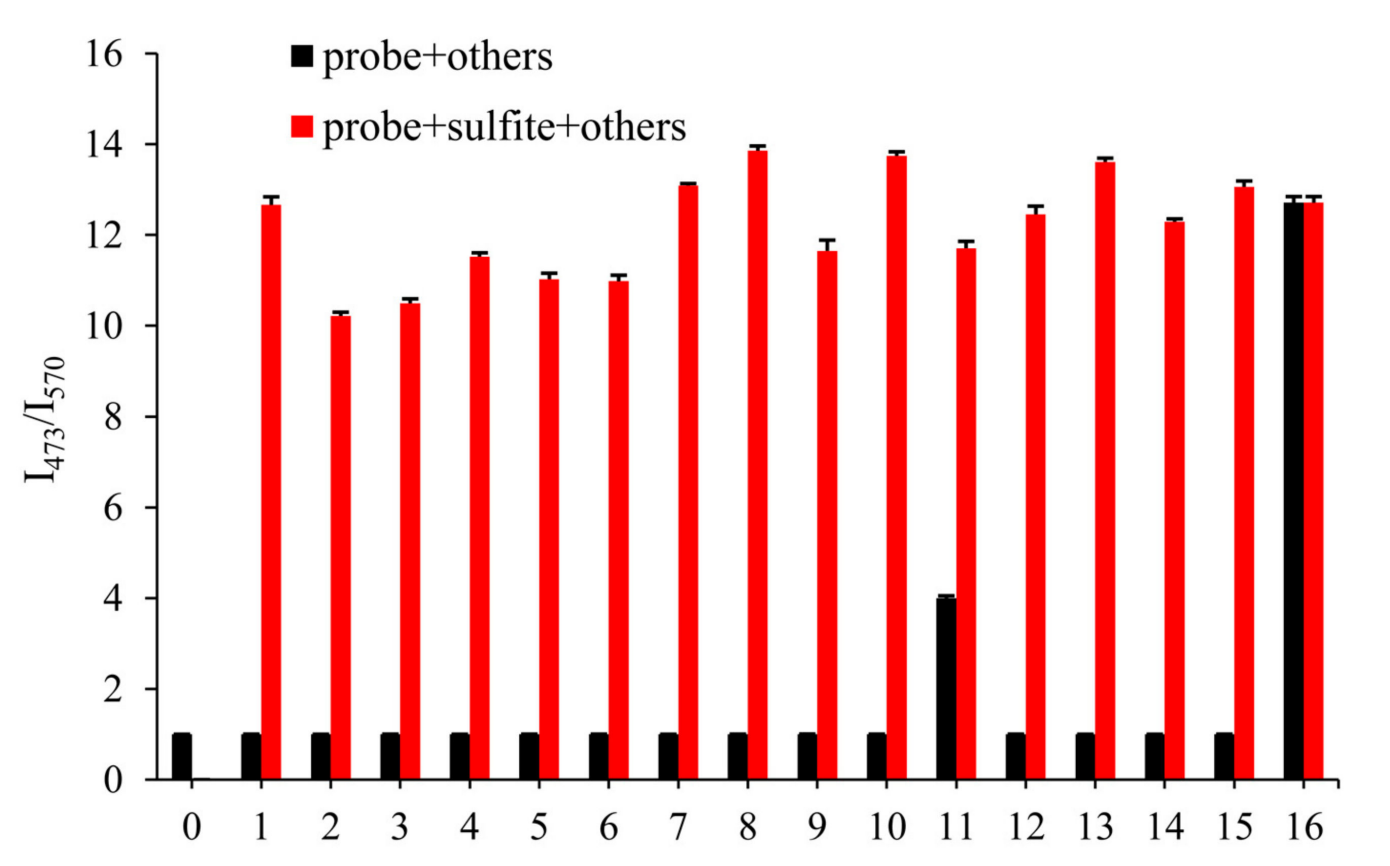
Fluorescence intensity of probe **EB** (10 μM) in PBS solution (pH 7.4) upon addition of various reactive sulphur and other anions (500 μM). 1. CO_3_^2−^, 2. HCO_3_^−^, 3. Ac^−^, 4. PO_4_^3−^, 5. HPO_4_^2−^, 6. H_2_PO_4_^−^, 7. Cl^−^, 8. Br^−^, 9. I^−^, 10. NO_2_^−^, 11. S^2−^, 12. SO_4_^2−^, 13. Hcy, 14. Cys and 15. GSH, 16. SO_3_^2−^. For λ_ex_ = 380 nm, λ_em_ = 473 slits: 5.0/5.0 nm; for λ_ex_ = 485 nm, λ_em_ = 570 nm slits: 10.0/10.0 nm.

The excellent selectivity of the probe played an essential role in practical application. In order to illustrate its anti-interference ability, we detected the fluorescence changes of probe **EB** (10 μM) with 500 μM other anions after 3 min, which aimed to prove no interference of the most common anions and biological thiols in the environment, including CO_3_^2−^, HCO_3_^−^, Ac^-^, PO_4_^3−^, HPO_4_^2−^, H_2_PO_4_^−^, Cl^−^, Br^−^, I^−^, NO_2_^−^, S^2−^, SO_4_^2−^, Hcy, Cys and GSH. As shown in Figure 2 (black), even 50 equivalents of the other species did not respond to the fluorescence ratio I_473_/_570_, but S^2−^ led to a slight increase of the fluorescence ratio I_473_/_570_, which may be influenced by the nucleophilicity of S^2−^. Meanwhile, in Figure 2 (red), the competition experiments meant that other analytes did not influence on detecting SO_3_^2−^ (500 μM). The results showed great selectivity of probe **EB** towards SO_3_^2−^, which indicated the excellent ability in the further application.

### 2.3. Time-Dependence and pH-Dependence in the Detection Process of Sulfite

**Figure 3:**
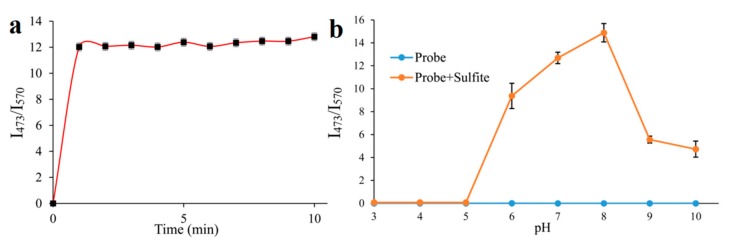
(**a**): Effect of time on the I_473_/I_570_ fluorescence intensity ratio of probe **EB** (10 μM) in PBS solution (pH = 7.4) upon addition of 500 μM SO_3_^2−^; (**b**): Effect of pH on the I_473_/I_570_ fluorescence intensity ratio of probe **EB** (10 μM) and probe **EB** upon addition of 500 μM SO_3_^2−^.

Time and pH are the pivotal factors, which could affect the application of the probe. Thus, with 500 μM SO_3_^2−^, we detected the fluorescent ratio (I_473_/_570_) changes of probe **EB** (10 μM) in different times (1–10 min) (Figure 3a). It showed that the fluorescent ratio (I_473_/_570_) of the probe tends to be stable in 1 min with the reaction of SO_3_^2-^ (Figure 3a), implying the probe could act as a “fast response” fluorescent probe for SO_3_^2−^ detection and may be used to timely sense SO_3_^2−^ in living cells. Furthermore, the effect of pH on the fluorescent ratio (I_473_/_570_) of the probe to SO_3_^2−^ was investigated ranging from pH 3 to 10. The fluorescent ratio (I_473_/_570_) of the probe was 0 without SO_3_^2−^ when the pH ranged from 3 to 10 (Figure 3b), suggesting the probe kept stable within a broad pH range. By adding SO_3_^2−^, the fluorescent ratio (I_473_/_570_) of the probe increased rapidly to the peak at pH 8 and then dropped modestly, which indicated that in the normal physiological ranges (pH = 7.4), the probe could reliably detect SO_3_^2−^/HSO_3_^−^.

As described above, the probe displayed excellent analytical property comparing with some other fluorescent probes of recent reports for the detection of SO_3_^2−^/HSO_3_^−^. The comparison data are listed in Table S1, indicating that the probe is promising for practical analysis.

### 2.4. Reaction Mechanism

**Figure 4:**
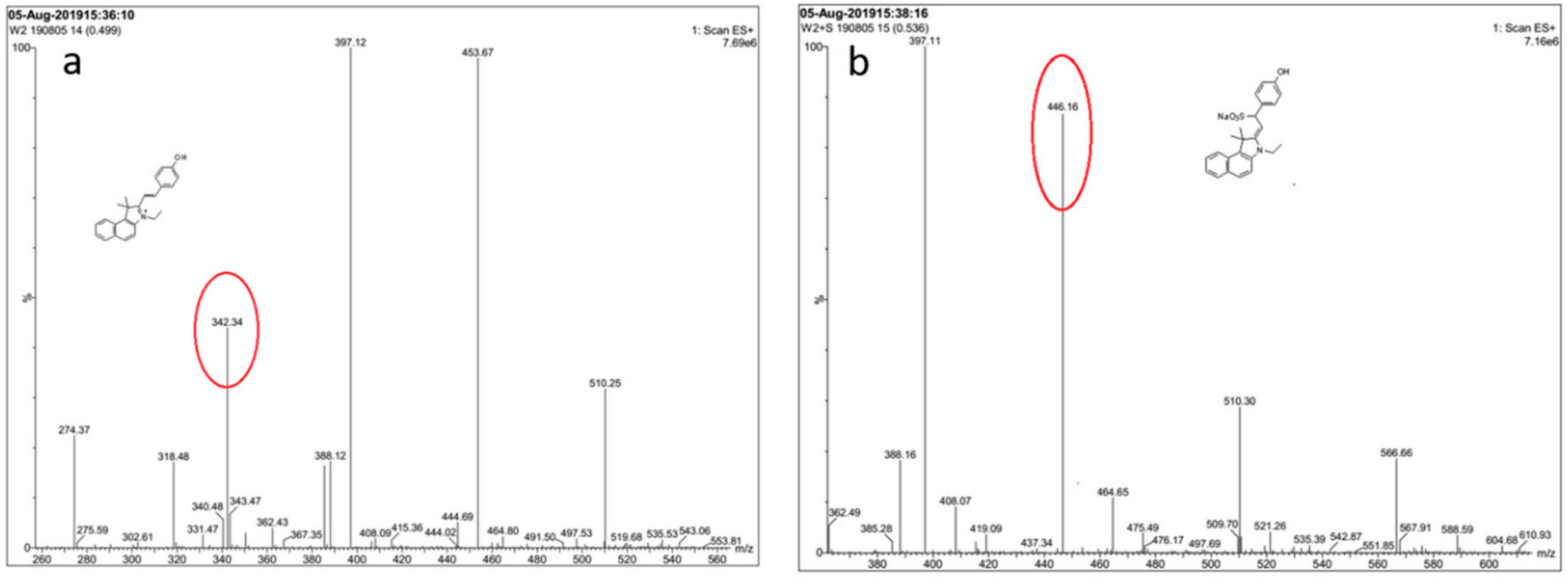
Mass spectrum of probe **EB** and the crude product of the probe with SO_3_^2−^. (**a**) Probe **EB** in MeOH. (**b**) Probe **EB** with Na_2_SO_3_ in MeOH: H_2_O (1:1).

In order to monitor the reaction mechanism, ESI-MS was used to inspect the reaction between probe **EB** and SO_3_^2−^. In the ESI-MS spectrum (Figure 4), the main peak at *m*/*z* = 342.34 was assigned to probe **EB** (calculated: [C_20_H_24_NO]^+^, 342.18). When Na_2_SO_3_ (10.0 equiv.) was added, the peak corresponds to probe-SO_3_^2-^ appeared at *m*/*z* = 446.16 (calculated: [C_24_H_24_NNaO_4_S + H]^+^, 446.13). On the basis of the above experiment results, the reaction mechanism of probe **EB** with SO_3_^2−^ was proposed and illustrated in Scheme 1, and it follows a 1,4-nucleophilic addition reaction of SO_3_^2−^ to the vinyl group of probe **EB**.

### 2.5. Sulfite Detection in Real Samples

In order to verify the practicability of the probe, we firstly investigated the sulfite levels in real samples by using probe **EB**, including tap water, river water, sugar water and chrysanthemum, which were dissolved and diluted in PBS solution (pH 7.4) into consistency for analysis. For comparison, a traditional titration method was used as validation. Two methods obtained almost consistent results (Table 1), which proved the practicability of probe **EB**. In addition, based on the above results, a standard addition method was used to detect sulfite in these real food samples. Adding an exact amount of SO_3_^2−^ (25, 50 and 100 μM) to the diluted sample, the I_473_/_570_ fluorescent ratio was measured. Excellent recovery ranged from 95.9% to 107.7% was observed in Table 1, suggesting the probe was practical and reliable for detecting sulfite in real samples.

### 2.6. Fluorescence Imaging in Living Cells

**Figure 5:**
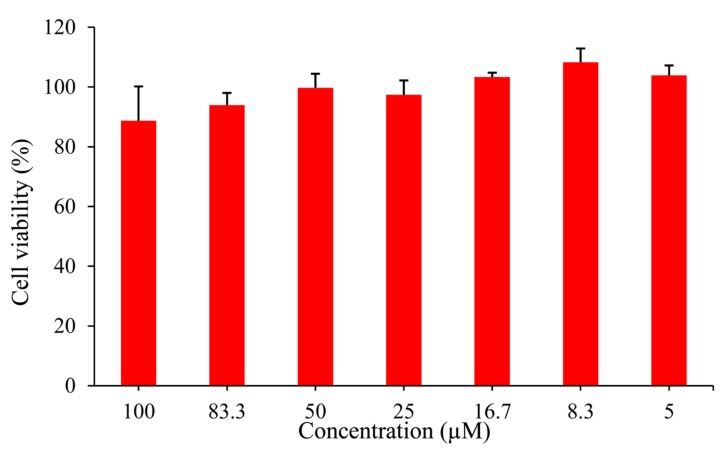
CCK8 assay of HepG2 cells incubated in the probe **EB** (0–100 μM) at 37 °C for 24 h.

The application of the probe for cell bioimaging was studied based on the results above. Cytotoxicity experiment was firstly incubated in HepG2 cells using Cell Counting Kit-8 (CCK8), and as shown in Figure 5, the survival rate of HepG2 cells was about to reach 90 % after incubating with 100 μM probe **EB** for 24 h, which suggested that the probe has low cytotoxicity for application in living cells.

**Figure 6:**
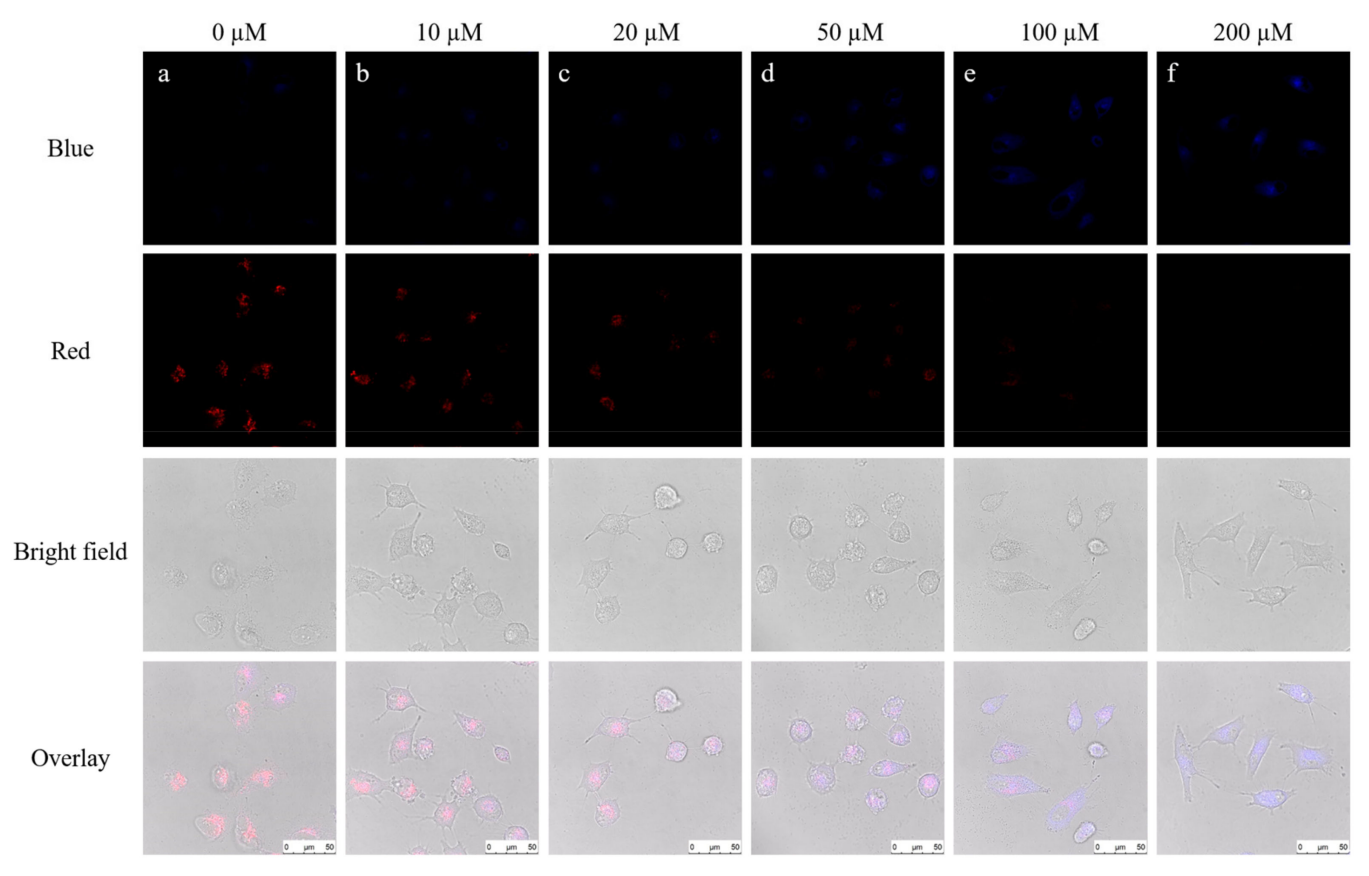
Imaging of SO_3_^2−^ in HepG2 cells by the probe. The cells were stained with (**a**) 10 μM probe **EB** for 2 h; (**b**) 10 μM SO_3_^2−^ for 2 h, 10 μM probe **EB** for 2 h; (**c**) 20 μM SO_3_^2−^ for 2 h, 10 μM probe **EB** for 2 h; (**d**) 50 μM SO_3_^2−^ for 2 h, 10 μM probe **EB** for 2 h; (**e**) 100 μM SO_3_^2−^ for 2 h, 10 μM probe **EB** for 2 h; (**f**) 200 μM SO_3_^2−^ for 2 h, 10 μM probe **EB** for 2 h; Blue channel: λ_ex_ = 405 nm; λ_em_ = 450–490 nm; Red channel: λ_ex_ = 488 nm; λ_em_ = 550–590 nm.

To verify the biological applications of the probe, we tried to explore potentialities about detecting SO_3_^2–^ in living cells. Firstly, we incubated the HepG2 with various concentrations of SO_3_^2–^ (0, 10, 20, 50, 100, 200 μM) for 2 h. After that quantitative probe (10 μM) was added into all cell groups waiting for 2 h, and monitoring tests were operated by the confocal laser-scanning microscopy. HepG2 cells showed a clear red profile after the incubation with probe (10 μM) for 2 h (Figure 6a), which suggested that the probe **EB** could permeate into HepG2 cells. While HepG2 cells were incubated with different concentrations of SO_3_^2–^ (0, 10, 20, 50, 100, 200 μM) for 2 h beforehand and then incubated with the probe **EB**. After 2 h, the fluorescence in the blue channel enhanced with the concentration of SO_3_^2−^ raised, but the fluorescence under the red channel degraded (Figure 6a–f). Finally, the HepG2 cells (Figure 6f) displayed strong blue fluorescence and weak red fluorescence. Therefore, the probe **EB** could be employed for the SO_3_^2−^ fluorescence imaging in living cells.

## 3. Materials and Methods

### 3.1. Materials and Instrumentation

The chemistry reagents (chemical grade) used in experiments was obtained from Sino Pharm Chemical Reagent Co. Ltd. (Shanghai, China). The real samples river water and tap water were obtained from the Shanghai University of Traditional Chinese Medicine, and sugar was purchased from a local supermarket (Shanghai, China). The chrysanthemum was purchased from Shanghai Kangqiao Chinese Medicine, grown in HangZhou, China. The analytical thin-layer chromatography (TLC) with pre-coated silica gel GF254 plates (Sino Pharm Chemical Reagent Co. Ltd., Shanghai, China) was used to detect any spots at 254 nm under UV light. ^1^H-NMR and ^13^C-NMR spectra were measured at 25 °C and referenced to tetramethyl silane (TMS) by a BRUKER AVANCE III spectrometer (Bruker, German). Electron ionization mass spectra (ESI-MS) was recorded on an Agilent 6460 triple quad LC-MS mass spectrometer (Agilent, Santa Clara, CA, USA). UV-Vis spectra and fluorescence spectra were recorded by Agilent 8454 UV-Vis spectrometer (Agilent) and Agilent G9800A fluorescence spectrophotometer (Agilent) respectively. Leica TCS-SP8 multiphoton illustrated the fluorescence images with the confocal microscope having a 63 × oil-immersion objective lens (Leica, Wetzlar, German).

### 3.2. Preparation of Probe **EB**

In a two-neck round-bottom flask, compound **1**, 3-ethyl-1,1,2-trimethyl-1*H*-benzo[e]indol-3-ium iodide (200.0 mg, 0.55 mM) and anhydrous ethanol (20 mL) were added. Subsequently, compound **2**, 4-hydroxybenzaldehyde (66.9 mg, 0.55 mM) was added to the mixture. This mixture reacted at 80 °C for 12 h. Then, the solution was disposed in a rotary vacuum desiccator and the solid was extracted with CH_2_Cl_2_/H_2_O. The organic layer was collected and dried via anhydrous Na_2_SO_4_. The crude product was purified with the CH_2_Cl_2_/MeOH eluent using the silica-gel column, see Supplementary Materials. Yield: 220 mg (87%). ESI/MS *m*/*z*: 342.34 [M]^+^; ^1^H-NMR (600 MHz, DMSO-d_6_) δ 10.86 (s), 8.52 (d, *J.* = 16.1 Hz), 8.42 (d, *J.* = 8.5 Hz), 8.30 (d, *J.* = 8.9 Hz), 8.25 – 8.16 (m), 8.12 (d, *J.* = 8.9 Hz), 7.81 (t, *J.* = 7.6 Hz), 7.72 (t, *J.* = 7.5 Hz), 7.53 (d, *J.* = 16.2 Hz), 7.00 (d, *J.* = 8.7 Hz), 4.90 – 4.72 (m), 2.03 (s), 1.50 (t, *J.* = 7.2 Hz). ^13^C-NMR (150 MHz, DMSO-*d*_6_) δ 196.44, 138.68, 137.51, 133.51, 131.20, 130.21, 128.89, 127.76, 127.73, 123.90, 113.66, 55.94, 43.80, 21.95, 14.10.

### 3.3. Preparation of Solutions and Spectra Measurements

The probe was dissolved in ethyl alcohol to prepare the 1 mM stock solution. The anion and amino acid solution such as CO_3_^2−^, HCO_3_^−^, Ac^-^, PO_4_^3−^, HPO_4_^2−^, H_2_PO_4_^−^, Cl^−^, Br^−^, I^−^, NO_2_^−^, S^2−^, SO_4_^2−^, Hcy, Cys, GSH and SO_3_^2−^ were all prepared by corresponding saline solution in purified water (10 mM). A small amount of stock probe **EB** (10 μM) and SO_3_^2−^ (500 μM) were transferred to a 5 mL volumetric flask, diluted with PBS (pH = 7.4) to volume as the test solution at room temperature, which was prepared to measure fluorescent spectra and UV-Vis absorption. The test solution should be shaken well and detected after 3 min. The same volume of SO_3_^2−^ solution could be replaced by various anion solutions mentioned above to detect interference using the same condition of spectra.

### 3.4. Measurements of Sulfite in Real Samples

Four kinds of samples: tap water, river water, sugar and chrysanthemum were prepared to detect sulfite respectively. Sugar and River water were diluted with PBS to a concentration of around 25 % (pH = 7.4) as the diluent solution. And the chrysanthemum sample was extracted with PBS solution (pH = 7.4) as the test solution (0.5 g/100 mL). Then transferred stock probe solution (10 μM) to a 5 mL volumetric flask, diluted with the diluent solution to volume. Incubated for 5 min at room temperature before detection. By the way, the recovery test also needed to add accurate SO_3_^2^^−^ (25, 50 or 100 μM) in water samples. Then, the emission intensities at 473 nm and 570 nm were detailed and the analysis results were also compared with that achieved by the titration method.

### 3.5. Methods for Fluorescent Quantum Yield

The fluorescent quantum yields of probe **EB** and probe **EB** after reaction with SO_3_^2^^−^ were detected using Rhodamine B (Φs = 0.89 in ethanol) as a standard. The Fluorescent quantum yields were determined based on the Equation:

Φ_a_ = Φ_st_ × (F_a_/F_st_) × (A_st_/A_a_) × (η_a_/η_st_)^2^

where Φ presents the fluorescent quantum yield, A presents the absorbance at the excitation wavelength of the probe and standard respectively, F presents the integrated fluorescence intensity of the sample and standard at their excitation wavelength, η presents the refractive index of solvent.

### 3.6. Limit of Detection

The detection limit (LOD) was calculated based on the fluorescence titration of the probe (10 μM) in the presence of SO_3_^2−^. The fluorescence intensity ratio of the probe was measured and the standard deviation of the blank measurement was achieved. The limit of detection was calculated according to the following formula: LOD = 3σ/k

LOD: detection limit; σ: the standard deviation of the fluorescence intensity ratio (I_473_/I_570_) of the probe scanning for 10 times; k: the slope of the line graph of fluorescence intensity ratio (I_473_/I_570_) and reactant concentration.

### 3.7. Cell Culture and Fluorescence Imaging

HepG2 cells came from the Chinese Academy of Sciences. The cells were cultured in Dulbecco’s modified Eagle’s medium (DMEM) added with 1% Penicillin-Streptomycin Solution (100×), 10% fetal bovine serum (FBS) at 37 °C under the atmosphere containing 5% CO_2_. Then transferred cells to confocal dishes and waited for 24 h to keep adaption.

To inspect the sensitivity of the probe, cells were incubated in 10 μM probe for 2 h at 37 °C with culture medium (90% DMEM, 10% FBS). To detect exogenetic sulfite, cells were pretreated with different concentrations of SO_3_^2^^−^ (0–200 μM) at 37 °C for 2 h and then incubated with the probe (10 μM) for 2 h. Before the detection achieved by the apparatus of Fluorescence Microscope and a 63× oil-immersion objective lens (Leica), cells needed to be washed by PBS for three times. The cells were stimulated by the intensity of wavelengths at 405 nm, 488 nm, and collected emission on 450–490 nm, 550–590 nm.

## 4. Conclusions

On the basis of the Michael addition reaction, we developed a new ratiometric fluorescent probe **EB** for SO_3_^2−^/HSO_3_^−^ detection under physiological pH, which exhibited a colorimetric and ratiometric response to SO_3_^2−^/HSO_3_^−^. It could demonstrate a quick response, high sensitivity and selectivity to SO_3_^2−^/HSO_3_^–^ in aqueous solution and real samples. The sensing mechanism of the interaction between the probe and SO_3_^2−^/HSO_3_^−^ was verified using ESI-MS. Moreover, it exhibited the excellent application of detecting SO_2_ derivatives in living cells with low cytotoxicity. All the results suggested this safe and harmless probe could be used conveniently.

## Figures and Tables

**Table 1:** Determination of SO_3_^2−^ in water samples using the probe **EB**.

Sample ^a^	SO_3_^2−^ Level Found (μM)	Added (μM)	Found (μM)	Recovery (%)
Tap water	0 ^b^	25.0	24.99 ± 0.55	99.9
	0 ^c^	50.0	48.87 ± 0.73	97.7
		100.0	95.92 ± 3.70	95.9
River water	1.8 ± 0.09 ^b^	25.0	25.9 ± 0.96	96.6
	1.5 ± 0.18 ^c^	50.0	52.4 ± 2.53	101.2
		100.0	102.3 ± 1.94	100.5
Sugar	0 ^b^	25.0	25.1 ± 1.31	100.4
	0 ^c^	50.0	52.9 ± 0.55	105.8
		100.0	104.8 ± 1.72	104.8
Chrysanthemum	16.8 ± 0.18 ^b^	25.0	43.73 ± 2.38	107.7
	15.9 ± 0.25 ^c^	50.0	69.2 ± 3.26	104.8
		100.0	115.9 ± 2.93	99.1

^a^ In the detection solutions, the concentration of sugar was 5.0 g/100 mL (pH 7.4); the River water was diluted 3 times with PBS solution (pH 7.4); the chrysanthemum sample was extracted with PBS solution (pH = 7.4) as the test solution (0.5 g/100 mL).By means of the three measurements(n = 3), we obtained the standard derivations. ^b^ Analysis for SO_3_^2-^ in water samples determined by the fluorescent probe. ^c^ Analysis for SO_3_^2−^ in water samples determined by a traditional titration method.

## Supplementary Materials

The following are available online. Figure S1. ^1^H-NMR spectrum of the probe in DMSO-d_6_; Figure S2. ^13^C-NMR spectrum of compound the probe in DMSO-d_6_; Figure S3. Mass spectrum of compound the probe. Table S1: Comparison of the probe for the detection of SO_3_^2−^/HSO_3_^−^.
