# Supplementary material for: A Ratiometric and Colorimetric Hemicyanine Fluorescent Probe for Detection of SO2 Derivatives and Its Applications in Bioimaging
Source: Molecules. 2019 Nov 5;24(21):4011. doi: 10.3390/molecules24214011 (PMC6865185; doi:10.3390/molecules24214011)
Supplement: Supplementary file 1 [file molecules-24-04011-s001.pdf]

Supplementary data

A ratiometric and colorimetric hemicyanine fluorescent probe for detection of SO_2 derivatives and its applications in bioimaging

Yan-Hong Qin[1†], Xiao-Yi Jiang[1†], Yuan-Fang Que[1], Jing-Yi Gu[1], Tong Wu[1], Ayinazhaer Aihemaiti[1], Ke-Xin Shi[1], Wen-Yu Kang[1], Bi-Ying Hu[1], Jin-Shuai Lan[1,2*], Yue Ding[1,2], Tong Zhang[1,2*]

1 School of Pharmacy, Shanghai University of Traditional Chinese Medicine, Shanghai 201203, China;

2 Experiment Center of Teaching & Learning, Shanghai University of Traditional Chinese Medicine, Shanghai 201203, China.

* Corresponding author. Tong Zhang; Jin-Shuai Lan

* E-mail: lanjinshuai_shut@126.com (J-S. L); zhangtongshutcm@hotmail.com (TZ)

† These authors contributed equally to this work.

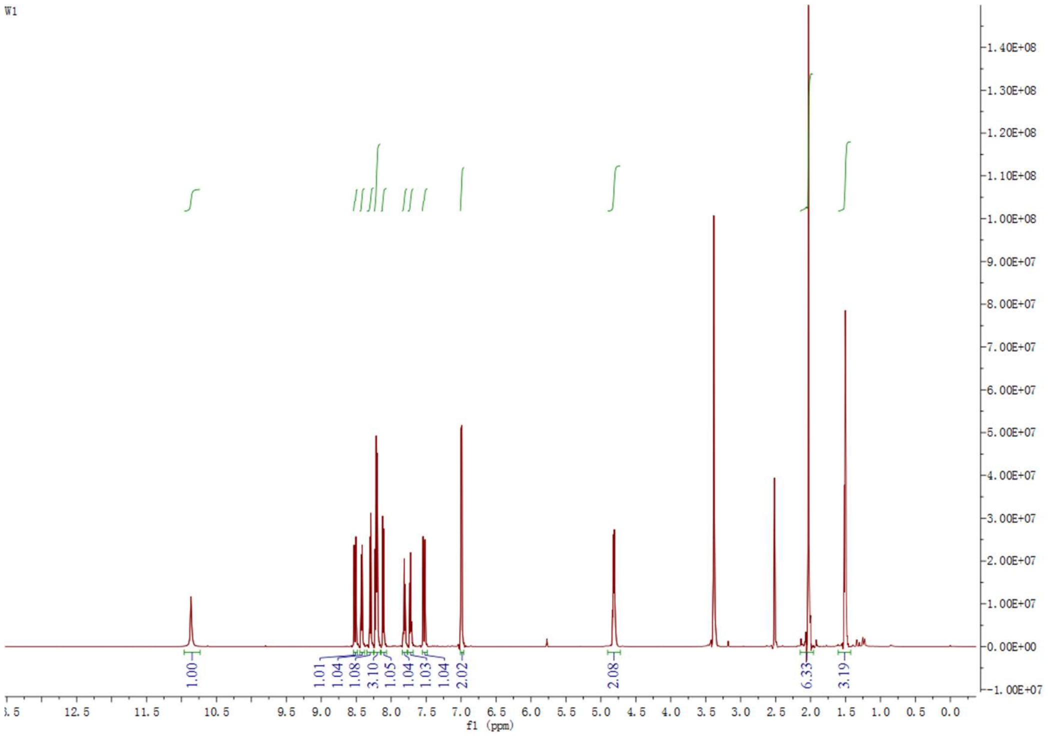

Fig. S1. ^{1}H-NMR spectrum of the probe in DMSO-d_6

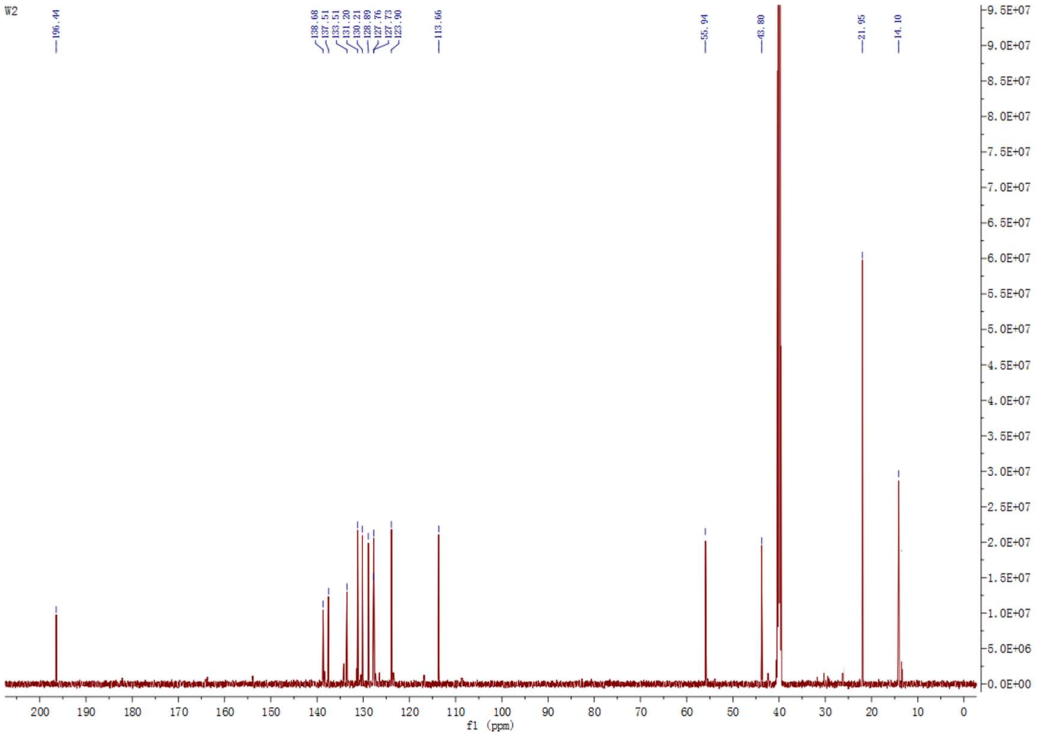

Fig. S2. ^{13}C-NMR spectrum of compound the probe in DMSO-d_6

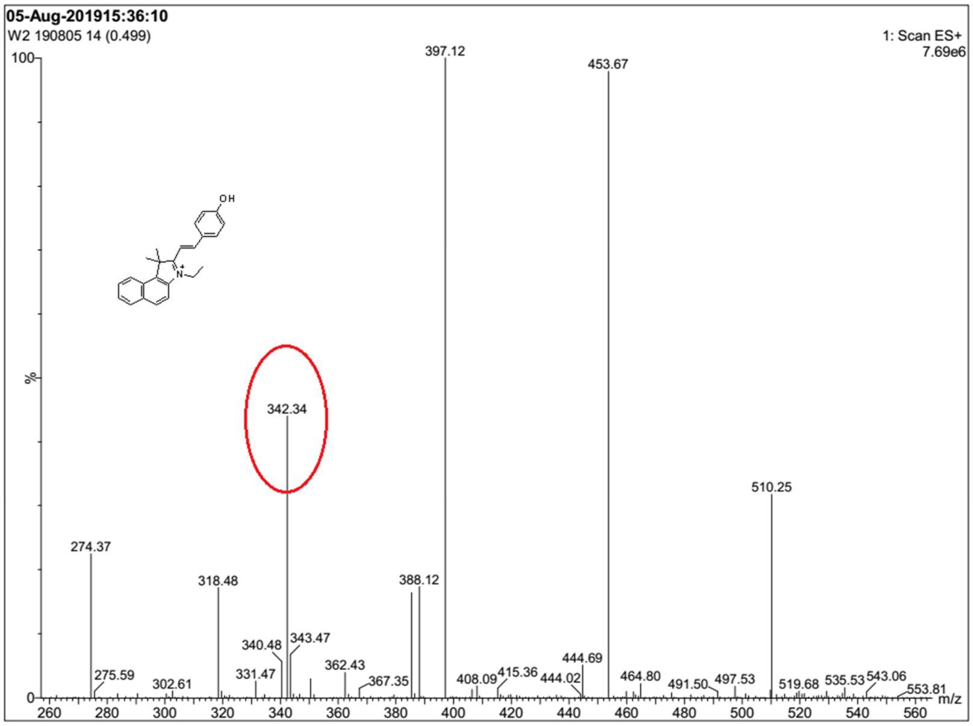

Fig. S3. Mass spectrum of compound the probe.

Table S1. Comparison of the probes for the detection of SO_3^{2-}/HSO_3^{-}.

Ref.	Probe	ratiometric probe	Response time	A linear range	Detection limit	Applied
S1		yes	90 s	0-10 μM	5.6 nM	real samples
S2		no	360 s	25-57 μM	71.4 nM	real samples
S3		no	240 s	0-50 μM	270 nM	HeLa cells

S4	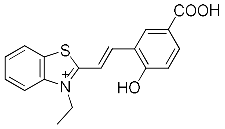	no	-	-	450 nM	293T cells
S5	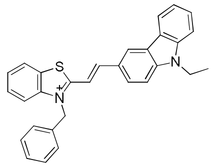	yes	35 s	0-14 μM	161 nM	MCF-7 cells/Daphnia magna
This work	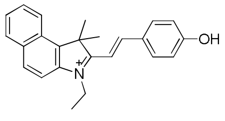	yes	60 s	0-60 μM	28 nM	real samples and HepG2 cells

References:

[S1] Sun, Y.; Zhao, D.; Fan, S.; Duan, L.; Li, R., Ratiometric fluorescent probe for rapid detection of bisulfite through 1,4-addition reaction in aqueous solution. *J Agric Food Chem* **2014**, 320 62, 3405-3409.

[S2] Li, Y.; Shi, L.; Zhang, Y.; Sun, G.; Sun, L.; Su, J., A simple dihydrophenazine-based chemosensor for the detection of sulfite with turn-on fluorescence. *Dyes and Pigments* **2019,** *160*, 794-798.

[S3] Wang, K.-P.; Lei, Y.; Sun, Y.; Zhang, Q.; Chen, S.; Zhang, Q.; Hu, H.-Y.; Hu, Z.-Q., Tetrahydro 5 helicene-based fluorescent probe for rapid and sensitive detection of bisulfite in living cells. *Sensors and Actuators B-Chemical* **2018,** *273*, 1487-1494.

[S4] Pan, X.; Zhong, Y.; Jiang, Y.; Zuo, G.; Li, J.; Dong, W., Reaction-based fluorescent sensor for detection of bisulfite through 1,4-addition reaction in water. *Materials Chemistry and Physics* **2018,** *213*, 83-88.

[S5] Li, H.; Zhou, X.; Fan, J.; Long, S.; Du, J.; Wang, J.; Peng, X., Fluorescence imaging of SO_2 derivatives in Daphnia magna with a mitochondria-targeted two-photon ratiometric fluorescent probe. *Sensors and Actuators B-Chemical* **2018,** *254*, 709-718.
